# Effects of Dentin Phosphophoryn-Derived RGD Peptides on the Differentiation and Mineralization of Human Dental Pulp Stem Cells In Vitro

**DOI:** 10.3390/biomedicines10112781

**Published:** 2022-11-01

**Authors:** Tubayesha Hassan, Youjing Qiu, Md Riasat Hasan, Takashi Saito

**Affiliations:** 1 Division of Clinical Cariology and Endodontology, Department of Oral Rehabilitation, School of Dentistry, Health Sciences University of Hokkaido, Tobetsu 061-0293, Hokkaido, Japan; 2 Stomatological Hospital of Xiamen Medical College, Xiamen Key Laboratory of Stomatological Disease Diagnosis and Treatment, Xiamen 361008, China

**Keywords:** dentistry, phosphophoryn-derived RGD peptide, odontoblast differentiation, dentin regeneration, vital pulp therapy

## Abstract

The purposes of this study were to investigate the in vitro effects of arginine-glycine-aspertic acid (RGD) peptides derived from human dentin phosphophoryn (DPP) on human dental pulp stem cell-proliferation, differentiation and mineralization, and to explore the mechanism of the peptides’ function. The 1 M concentration of soluble DPP-derived RGD peptides, RGD-1, RGD-2 and RGD-3 were coated onto non-tissue-culture polystyrene plates, and human dental pulp stem cells (hDPSCs) were cultured on them to examine the effects of the peptides on hDPSCs. In addition, 1 M arginine-alanine-aspertic acid (RAD) peptides were used as the control. Cell proliferation of hDPSCs was promoted by all three RGD peptides. All three RGD peptides had significantly higher alkaline phosphatase (ALP) activity compared to the control. RGD-3 induced the highest ALP activity compared to the control. RGD-3 also significantly promoted the mRNA expression of the following genes: 1.69-fold in *dentine matrix protein-1* (*DMP-1*), 1.99-fold in *dentine sialophosphoprotein* (*DSPP*), 1.51-fold in *ALP*, and 2.31-fold in *bone sialoprotein* (*BSP*), as compared to the control group. Mineralization of hDPSCs was accelerated by all three RGD peptides, RGD-3 in particular. The MAPK p38 inhibitor SB202190 inhibited the effect of RGD-3 to a level comparable to the control, observed in both ALP activity assay and Arizarin red S (ARS) staining. It suggests that the p38 pathway may be responsible for eliciting the differentiation and mineralization effects of DPP-derived RGD peptides in the hDPSCs. The mRNA expression levels of the integrins *ITGA1-5*, *ITGA7*, *ITGB1* and *ITGB3* were significantly upregulated. Among them, expression of *ITGA5* was promoted 1.9-fold, *ITGA7* 1.58-fold, *ITGB1* 1.75-fold and *ITGB3* 1.9-fold compared to the control. It suggests the possible involvement of these integrin channels in different subunit combinations facilitating signal transduction for differentiation of hDPSCs into odontoblasts. As conclusions, human DPP-derived RGD peptides RGD-1, RGD-2 and RGD-3 promoted the proliferation, differentiation and mineralization of hDPSCs in vitro. Among the three peptides, RGD-3 had the most significant effects. It is also suggested that RGD-3 binds to integrin receptors on the surface of hDPSCs and regulates the odontogenic gene expression and differentiation via activation of p38 of MAPK pathway. DPP-derived RGD-3 may be a promising choice in the formulation of a novel material for vital pulp therapy to induce dental pulp stem cells into odontoblasts and form reparative dentin on the exposed pulp tissue.

## 1. Introduction

A healthy dental pulp is crucial in the maintenance of the structural and functional soundness of a tooth. In cases of tooth devitalization due to a deep carious lesion or trauma, newly differentiated odontoblast-like cells migrate toward the site of injury and form reparative dentine to protect pulp tissue from the harmful stimuli. In direct pulp capping, a protective agent is applied to injured pulp tissue to promote pulp healing and preserve the integrity of the tooth. Calcium hydroxide [Ca(OH)_2_] and mineral trioxide aggregate (MTA) are the current standard materials used for this treatment [1,2]. However, due to their limitations, the clinical performances of these materials are unsatisfactory [3,4,5]. Therefore, researchers continue to develop a novel biocompatible pulp capping material with the goal of regenerating dentin of significantly improved quality.

Dentin phosphophoryn (DPP) is the most abundantly present noncollagenous protein in dentine, and is highly acidic and anionic in nature due to the presence of copious amounts of aspartic acid and phosphoserine in its amino acid structure. Studies have shown that it binds to calcium ions [6] and mediates reparative dentine formation of improved quality compared to Ca(OH)_2_ [7]. It is a cleaved product of dentin sialophosphoprotein (DSPP), and the absence of DSPP has been associated with dentinogenesis imperfecta type III [8]. According to a study, dentin sialoprotein (DSP) plays a role in regulating the initiation of dentine mineralization and DPP in the maturation of mineralized teeth [9]. These findings suggest the importance of DPP in dentinogenesis during developmental stage. Several studies have been conducted to investigate the role of phosphoproteins in the induction of dentine mineralization. It was reported that DPP promotes mineralization of bone and dentine [6,10]. DPP induces the formation and growth of hydroxyapatite crystals in dentine when covalently cross-linked to collagen fibrils [11]. In addition, DPP has acted as a marker for pulp cell differentiation into odontoblasts [12]. It was found that phosphophoryn regulates gene expression in mesenchymal stem cells via the integrin/mitogen activated protein kinase (MAPK) pathway [13]. Additionally, DPP elicits signalling via activation of the Smad pathway [14].

The amino acid chain of human DPP contains a species-conserved arginine-glycine-aspertic acid (RGD) cell attachment domain and a repeated Asp-PSer-PSer sequence. The RGD domain has potential effect in stimulating specific cellular responses, as DPP has been shown to mediate cell adhesion and migration by initiating integrin-mediated signalling on the surface of pulp cells via its RGD motif in vitro [15]. The osteogenic effects of several RGD peptides from human DPP were investigated. Among the various peptides, RGD-1, RGD-2 and RGD-3 promoted human mesenchymal stem cell differentiation into osteoblasts [16]. In a later study, human DPP-derived RGD-peptides showed similar effects on the differentiation and mineralization of the rat odontoblast-like cell line MDPC-23. RGD-3 showed remarkable results among the three peptides [17]. Another study [18] observed the role of DPP on the differentiation of MDPC-23 cells. From the upregulation of odontogenic gene expressions, it was suggested that DPP might bind integrin receptors on the surface of MDPC-23 cells via its RGD motif.

However, the specific role of the DPP-derived RGD domain has not yet been studied as extensively as the DPP protein. As the background of the present study, a previous study [16] experimented with various configurations of the amino acid sequence of the RGD domain in human DPP and named them chronologically: RGD-1 (SESDNNSSSRGDASYNSDES), RGD-2 (ANSESDNNSSSRGDA), RGD-3 (SRGDASYNSDESKD), RGD-4 (DNNSSSRGDASYNSD), RGD-5 (SSSRGDASY) and a mutant peptide RGD-6 (SESDNNSSSRADASYNSDES), which was named arginine-alanine-aspertic acid (RAD)-1 in the later study [17]. Among the peptides that promoted differentiation of mesenchymal stem cells into osteoblasts were RGD-1, RGD-2 and RGD-3. Based on the results of this study, the possible functional significance of these three RGD peptides and their ability to induce proliferation, differentiation and mineralization of odontoblast-like cells were evaluated in one study [17]. RGD peptides were immobilized onto modified tissue culture polystyrene surfaces, and MDPC-23 cells were cultured onto the surfaces. It was reported that RGD peptides, especially RGD-3-0.5 mg/mL, have significant effect on the differentiation and mineralization of MDPC-23 cells. In the present study, the configurations of RGD-1, RGD-2 and RGD-3 in these previous two studies were used, as well as the peptide named RAD-1 [17].

Most of the studies conducted regarding DPP have been conducted on animal cells like rat odontoblast-like cells mdpc-23, thus making the results less suitable for human cells. Moreover, the MDPC-23 cells are of terminally differentiated odontoblastic lineage; while the hDPSCs are undifferentiated and can differentiate into some other lineage than odontogenic. Although there have been several studies done with RGD peptides, they were not derived from human DPP. Therefore, the activity of human recombinant DPP, including the specific role of the RGD domain, needed to be established. The aims of this study were to investigate the in vitro effects of human DPP-derived RGD peptides on the proliferation, differentiation and mineralization of hDPSCs, and to explore the mechanism of the peptides’ function in vitro.

## 2. Materials and Methods

### 2.1. Coating of RGD Peptides and RAD Peptides

The DPP-derived RGD peptides; RGD-1 (SESDNNSSSRGDASYNSDES), RGD-2 (ANSESDNNSSSRGDA), RGD-3 (SRGDSASYNSDESKD) and RAD peptides; RAD-1 (SESDNNSSSRADASYNSDES), RAD-2 (ANSESDNNSSSRADA) and RAD-3 (SRADSASYNSDESKD) were custom-synthesized by GL Biochem Ltd. (Shanghai, China) and stored at −30 °C until use. The effect of coating of human DPP-derived RGD peptides on non-tissue culture treated polystyrene (non-TCPP, Corning, NY, USA) plates was analysed. The effect of RAD peptides was also investigated, which were synthesized by replacing the RGD domain with the corresponding RAD peptide sequences and coated on non-TCPP plates. Briefly, RGD peptide powder was dissolved in distilled water (dH_2_O, manufacture) at a 1 M concentration (0.5 mg/mL RGD-1, 0.36 mg/mL RGD-2 and 0.36 mg/mL RGD-3), spread onto non-TCPP plates and then dried at room temperature for two days. RAD-peptides (1 M, 0.52 mg/mL RAD-1, 0.36 mg/mL RAD-2 and 0.37 mg/mL RAD-3) were also coated on plates following a similar technique. dH_2_O was used as a negative control. In experiment two, RGD-3-coated groups were exposed to certain MAPK inhibitors and their effect was observed. In experiment three, the gene expression levels of integrins in RGD-3-coated group were investigated.

### 2.2. Cell Culture

Human dental pulp stem cells (hDPSCs) (catalog number: 5025; lot number: 0000361427) were purchased from LONZA (Walkersville, MD, USA). The hDPSCs were cultured in Dulbecco’s modified Eagle’s medium (DMEM, Sigma-Aldrich, St. Louis, MO, USA) supplemented with 10% fetal bovine serum (FBS, Gibco, Grand Island, NY, USA). All media were supplemented with 50 units/mL penicillin and 50 µg/mL streptomycin (Gibco). Culture media was changed every four days. Cell passages from 3 to 5 were used in this study. Cells were inoculated at a density of 4 × 10^3^ cells/well in 96-well plates, 2 × 10^4^ cells/well in 12-well plates, and 3 × 10^4^ cells/well in 6-well plates. Cells were cultivated at 37 °C under humidified 5% CO_2_ and 95% air atmospheric conditions.

### 2.3. Cell Proliferation Assay

Cell Counting Kit-8 (CCK-8) was purchased from Dojindo (Kumamoto, Japan). For the cell viability assay, RGD peptides (1 M and their different diluted concentrations) were coated on 96-well microplates and dried for 2 days at room temperature. hDPSCs were cultured in DMEM supplemented with 10% FBS, 50 units/mL penicillin, and 50 μg/mL streptomycin for 4 days. Concentrations were as follows: 1, 0.5, 0.05 and 0.005 mg/mL for RGD-1, 0.72, 0.36, 0.036 and 0.004 mg/mL for RGD-2, and 0.72, 0.36, 0.036 and 0.004 mg/mL for RGD-3. dH_2_O was used as a control. On day 5, CCK-8 reagent was added to each well (10 µL/well) and incubated with the cells for 80 min in the incubator. The absorbance of lysates was measured using a microplate reader at 450 nm.

### 2.4. Cell Morphology Observation

hDPSCs were seeded at 3 × 10^4^ cells/well in 6-well microplates. RGD-coated groups (1 M of RGD-1, RGD-2 and RGD-3) were compared with cells in the control group. Cells were visualized at specific time intervals under phase contrast microscopy (Olympus, Tokyo, Japan) on days 1, 3 and 5.

### 2.5. Alkaline Phosphatase (ALP) Activity Assay

Cells were seeded at 3 × 10^4^ cells/well in 6-well microplates and cultured in DMEM supplemented with 10% FBS, 50 units/mL penicillin and 50 μg/mL streptomycin for six days. Then, 10 mM glycerol-2-phosphate disodium salt n-hydrate (β-GP, Wako, Osaka, Japan) and 50 μg/mL L-ascorbic acid phosphate magnesium salt n-hydrate (AA, Wako) were added on day 7 when the cells reached confluence. For experiment one, non-TCPP plates were coated with 1 M RGD-1, RGD-2 and RGD-3 and 1 M RAD-1, RAD-2 and RAD-3. After incubation for the prescribed number of days, an ALP activity assay was performed on days 8, 15 and 22. Alkaline phosphatase (ALP) assay kit was purchased from Wako (Osaka, Japan). The Pierce BCA protein assay kit was purchased from Thermo Fisher Scientific (Rockford, IL, USA).

Cells were collected and lysed with 0.1% Triton-X-100 (Sigma-Aldrich) in dH_2_O. Then lysates were sonicated in a sonicator (Bioruptor^®^, Diagenode, Seraing, Belgium) on ice for 10 min and centrifuged at 12,000 rpm at 4 °C for 15 min, and then the supernatants were collected. ALP activity and protein quantification were determined according to the manufacturers’ instructions. One unit of ALP activity was defined as the release of 1 nmol *p*-nitrophenol per minute at pH 9.8 and 37 °C. The relative activity was determined as follows: units/μg protein = activity (units/μL)/protein concentration (μg/μL). Absorbance was measured at 405 nm for ALP activity and 570 nm for protein quantification using a microplate reader. Values were averaged from triplicate samples.

### 2.6. Real-Time Quantitative RT-PCR

Cells were seeded at 3 × 10^4^ cells/well in 6-well microplates and cultured in DMEM containing 10% FBS, 50 units/mL penicillin and 50 μg/mL streptomycin for six days. Then, 10 mM β-GP and 50 μg/mL AA were added on day 7 when the cells reached confluence. For experiment one, non-TCPP plates were coated with 1 M RGD peptides and 1 M RAD peptides. 

On day 21, total RNA was isolated using Trizol^®^ (Invitrogen, Carlsbad, CA, USA). The isolated RNA was pelleted, washed in 75% ethanol, and re-suspended in nuclease-free water. A NanoDrop ND-1000 spectrophotometer (Thermo Fisher Scientific) was used to spectroscopically measure the RNA concentration of each sample. Afterward, one microgram of RNA concentration was reverse-transcribed into complimentary DNA (cDNA) using the M-MLV (Invitrogen) reverse transcriptase in a 20 μL reaction system (which included 5× reaction buffer, 500 μg/mL oligo dt, 2.5 mmol/L dNTP mixture, 40 unit/μL RNase inhibitor, 0.1 mol/L DTT, and 1 μL M-MLV) according to the manufacturers’ instructions. The resultant cDNA was used for real-time RT-PCR.

To quantify odontogenic gene expressionand gene expression of integrin subunits in hDPSCs, real-time RT-PCR was performed on a LightCycler™ Nano (Roche, Basel, Switzerland). Specific primers for *dentine matrix protein-1* (*DMP-1*), *dentine sialophosphoprotein* (*DSPP*), *ALP*, *runt-related transcription factor-2* (*Runx-2*), *bone sialoprotein* (*BSP*), *osteopontin* (*OPN*), *ITGA1-8*, *ITGB1* and *ITGB3* were used. Relative gene expression was calculated using the comparative 2^−∆∆Ct^ method. *hGAPDH* was used as an internal standard to normalize mRNA expression levels. The sequences of primers used for are presented in Table 1 and Table 2.

### 2.7. Arizarin Red S (ARS) Staining

To observe and quantify calcific deposition of hDPSCs, alizarin red S (ARS) staining and the cetylpyridinium chloride (CPC) extraction method were used. Cells were spread at 3 × 10^4^ cells/well in a 6-well microplate and cultured in DMEM supplemented with 10% FBS, 50 units/mL penicillin and 50 μg/mL streptomycin. Then, 10 mM β-GP, 50 μg/mL AA, and 100 nmol/L dexamethasone (Dex, Sigma-Aldrich) were added on day 7 when the cells reached confluence. ARS staining was performed on days 29 and 31.

Cells were fixed with 10% formalin neutral buffer solution (Wako) for 20 min, and washed again with phosphate buffered saline (PBS, pH 7.4, Gibco). ARS solution (1%, pH 4.1, Wako) was added to the cell monolayer, and after 10 min, the staining solution was removed. Then, the cells were washed three times with distilled water and washed thoroughly with PBS to remove the nonspecific background stain. Photographs were taken by a digital imaging system (Funakoshi, Tokyo, Japan). After staining with ARS, CPC (10%, *w*/*v*, in distilled water, Sigma-Aldrich) was added to each dish (1 mL per dish) and incubated with the cells for 1 h at 37 °C. After incubation, the transparent CPC solution turned into purple, was diluted by four times in additional CPC solution (10%) and transferred to a 96-well plate (80 μL per well). Absorbance was read at 570 nm.

### 2.8. Effects of MAPK Inhibitors

To investigate the effects of the inhibitors on cell differentiation, cells were seeded at 2 × 10^4^ cells/well in a 1 M RGD-3-coated 12-well microplate. To check the effects of the inhibitor on cell mineralization, cells were seeded at 2 × 10^4^ cells/well in a 1 M RGD-3-coated 6-well microplate. Cells were challenged with 20 μM of one of three MAPK pathway inhibitors on day 6 in serum-free DMEM for 2 h, SP600125 (Cell Signaling Technology, Danvers, MA, USA) for c-Jun N-terminal kinase (JNK), SB202190 (Cell Signaling Technology) for p38 and PD98059 (Cell Signaling Technology) for extracellular signal-regulated kinase (ERK), respectively. Then, the medium was changed to DMEM supplemented with 10% FBS, 50 units/mL penicillin and 50 μg/mL streptomycin, and 10 mM β-GP and 50 μg/mL AA were added on day 7 when the cells reached confluence. ALP activity assays were performed on days 8 and 22. ARS staining was performed on day 31.

### 2.9. Statistical Analysis

All experiments were carried out in triplicate, and the results are expressed as the mean ± standard deviation. Data were subjected to one-way ANOVA (analysis of variance) and post-hoc Tukey HSD (honestly significant difference) for statistical analysis with significance level at *p* < 0.05.

## 3. Results

### 3.1. Cell Morphology Observation

**Figure 1:**
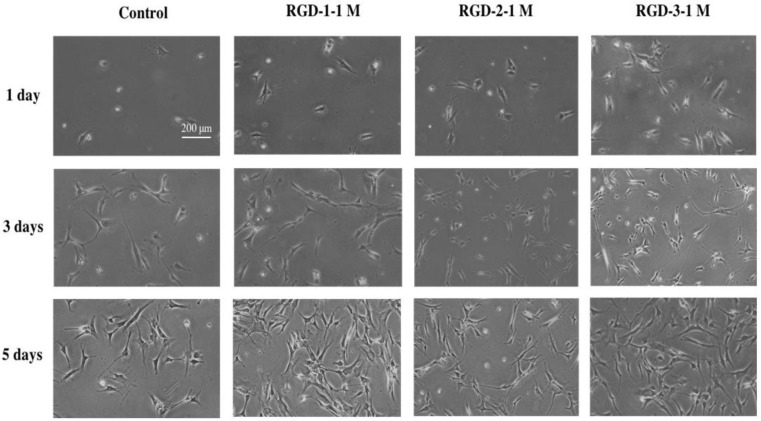
Cell morphology observation. hDPSCs were spread on non-TCPP plates. Plates were coated with 1 M soluble RGD-1, RGD-2, or RGD-3 in the experiment group, dH_2_O was used as a control. Cell morphology was observed on days one, three, and five. The scale bar in the control group image on day one applies to all panels (bar = 200 µM).

Cell morphology was observed via light microscopy images, which are shown in Figure 1. It can be clearly observed that hDPSCs grown on RGD-coated surfaces displayed an extended morphology. From day 1 images, it is visible that the number of attached cells is higher in RGD groups than in the control. Cells in the RGD groups were successfully attached, spread, and adopted an extended shape, and the cells continued to grow during later observation time points of observation in all three RGD groups. In the control group, most cells showed a spherical morphology and extended incompletely on day 1. On day 3 and day 5 images, the cell number was higher in the RGD-coated groups than in the control. Cell number was counted with a haemocytometer, and was significantly higher in all the RGD peptide groups: mean: RGD-1 (11.74 ± 0.41 × 10^4^), RGD-2 (9.96 ± 0.27 × 10^4^) and RGD-3 (11.92 ± 0.31 × 10^4^) values compared to the control (4.77 ± 0.45 × 10^4^) on day 5.

### 3.2. Cell Proliferation

**Figure 2:**
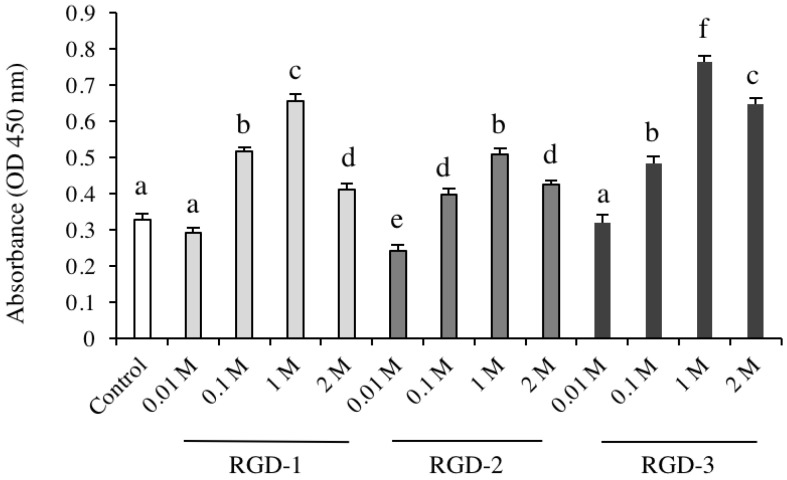
Effects of DPP-derived RGD peptides on the proliferation of hDPSCs. Plates were coated with different concentrations of soluble RGD-1, RGD-2 or RGD-3 in the experiment groups, and in the control group, plates were coated with dH_2_O. After four days of culture, absorbance was measured using a microplate reader, OD = 450 nm. Significant differences were observed between the control and RGD-coated groups. RGD at a concentration of 1 M (0.5 mg/mL RGD-1, 0.36 mg/mL RGD-2 and 0.36 mg/mL RGD-3) maximized cell proliferation. *n* = 6, different lowercase characters denote significant differences compared to the control (*p* < 0.05).

hDPSCs proliferation was investigated in response to various concentrations of RGD peptides using CCK-8 assays. In all three RGD groups, cell proliferation was significantly promoted compared to the control. Among all the concentrations that were tested, cell proliferation was maximized at a 1 M concentration for each peptide (Figure 2).

### 3.3. ALP Activity

**Figure 3:**
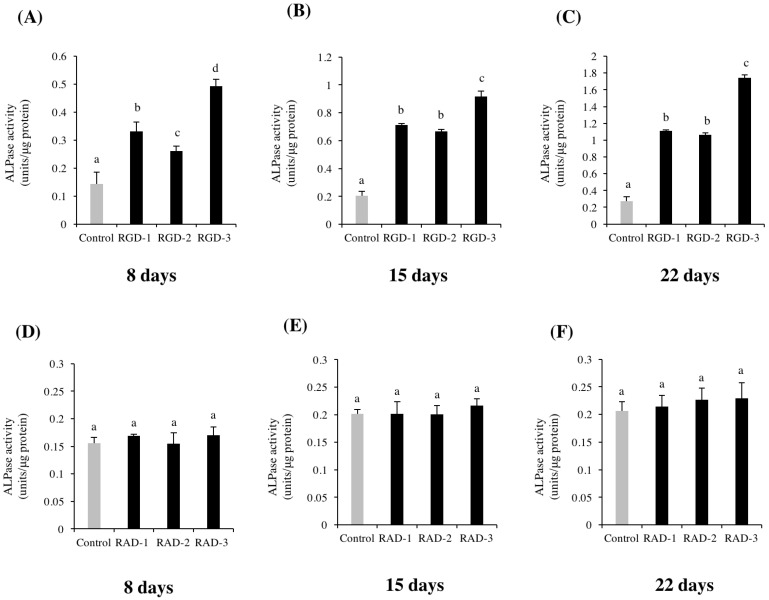
Effects of DPP-derived RGD peptides and RAD peptides on the ALP activity of hDPSCs. hDPSCs were spread onto non-TCPP plates coated with 1 M RGD peptides (RGD-1, RGD-2 and RGD-3) or dH_2_O (control). ALP activity was assessed on days 8 (**A**), 15 (**B**) and 22 (**C**). Cells were spread on to 1 M RAD peptide-coated plates (RAD-1, RAD-2 and RAD-3) and ALP activity was assessed on days 8 (**D**), 15 (**E**) and 22 (**F**). The relative ALP activity in all three RGD peptides were higher than in the control group. RAD peptides showed comparable results to the control group. *n* = 3, different lowercase characters denote significant differences compared to the control (*p* < 0.05).

An ALP activity assay was performed to detect hDPSC differentiation toward odontogenic lineages. Figure 3A–C illustrate that the relative ALP activity in the RGD-coated groups was significantly increased compared to that in the control group on all days evaluated. In experiment one, all three RGD peptides showed significantly higher ALP activity than the control on all days evaluated. The ratios of ALP activity in the RGD-coated groups divided by that in the control group were 2.57 on day 8, 3.72 on day 15, and 4.08 on day 22. RGD-3 induced the highest ALP activity on all days (0.5 ± 0.03 units/µg protein, 0.92 ± 0.04 units/µg protein, and 1.74 ± 0.04 units/µg protein, respectively). However, the relative ALP activity in the RAD-coated groups was comparable to that in the control group on all days, as illustrated in Figure 3D–F.

### 3.4. Real-Time RT-PCR

**Figure 4:**
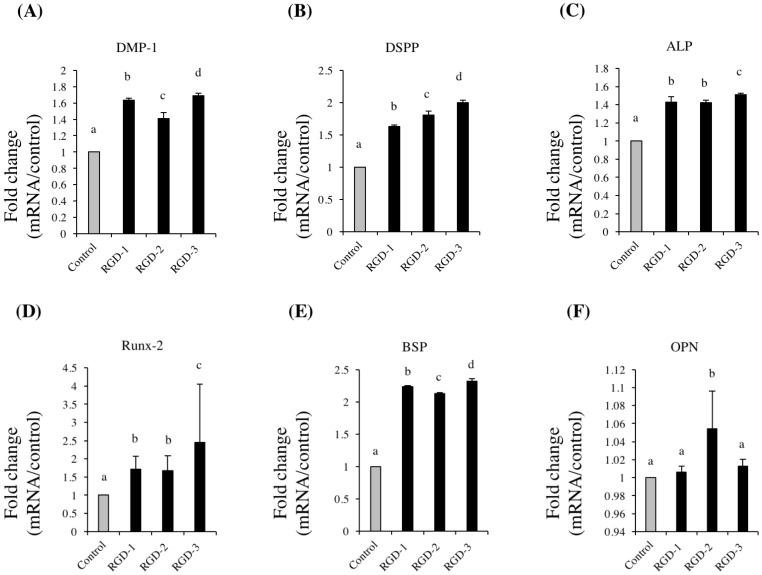
Effects of RGD peptides on the mRNA expression of odontogenic differentiation markers. hDPSCs were spread onto non-TCPP plates coated with 1 M RGD peptides (RGD-1, RGD-2 and RGD-3) or dH_2_O (control). Real time RT-PCR was performed on day 21. The mRNA expression of *hDMP-1* (**A**), *hDSPP* (**B**), *hALP* (**C**) and *hBSP* (**E**) was significantly upregulated by RGD peptides. The differences in hRunx-2 (**D**) and *hOPN* (**F**) mRNA expression were less evident. *n* = 3, different lowercase characters denote significant differences compared to the control (*p* < 0.05 vs. control).

Real-time RT-PCR was performed to investigate the effects of human DPP-derived RGD peptides on the mRNA expression of odontogenic differentiation marker genes. All three RGD peptides significantly upregulated of the expression of the target genes, including *DMP-1*, *DSPP*, *ALP*, *Runx-2* and *BSP*, as compared to the control group (Figure 4A–F). RGD-3 induced the highest mRNA expression among the three RGD peptides: 1.69-fold in *DMP-1*, 1.99-fold in *DSPP*, 1.51-fold in *ALP* and 2.31-fold in *BSP*, as compared to the control group.

**Figure 5:**
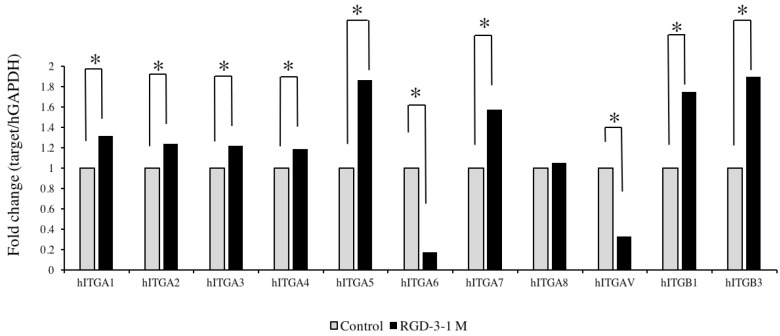
Effects of RGD-3 on the mRNA expression of various integrin subunit genes. Control represents cells cultured in the presence of β-GP, AA, and dH_2_O, RGD-3 group represents cells cultured in β-GP, AA, and 1 M RGD-3. All the integrins showed enhanced mRNA expression compared to the control. Among them, the mRNA expression of *hITGA5*, *hITGA7*, *hITGB1* and *hITGB2* exhibited strong promotion. *n* = 3. ∗ Denotes significant difference when compared to the control (*p* < 0.05 vs. control).

The mRNA expression of integrin subunit genes was measured to investigate the cell surface integrins that may potentially bind to the RGD-3. The integrins *ITGA1*, *ITGA2*, *ITGA3*, *ITGA4*, *ITGA5*, *ITGA7*, *ITGB1* and *ITGB3* had significantly upregulated mRNA expression levels (Figure 5). Among them, *ITGA5* expression was promoted 1.9-fold, *ITGA7* was promoted 1.58-fold, *ITGB1* was promoted 1.75-fold and *ITGB3* was promoted 1.9-fold, as compared to that in the control group.

### 3.5. ARS Staining

**Figure 6:**
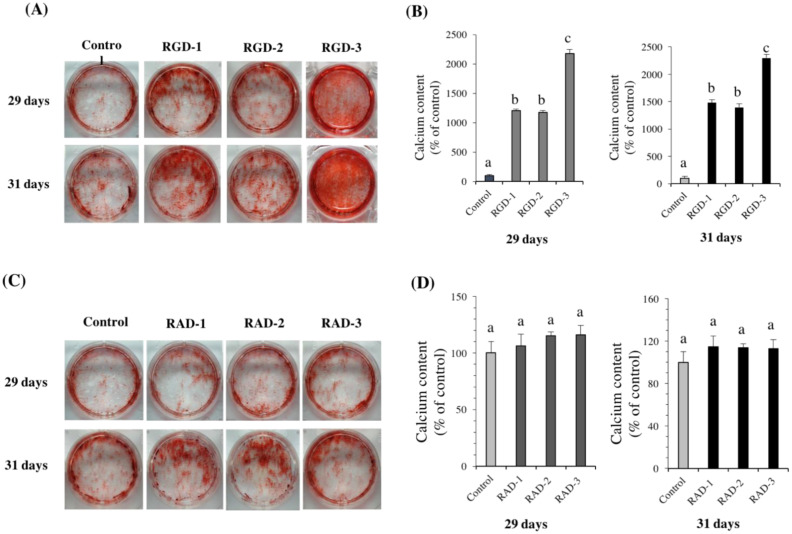
Effects of RGD peptides on hDPSC mineralization. (**A**) Photographs of mineral nodules formed after 29 and 31 days of culture in experiment one. (**B**) CPC quantification of mineralized product staining intensity in (**A**). The RGD-coated groups exhibited a strong promotion of calcific deposition in hDPSCs than the control group on both days 29 and 31. On both days, RGD-3 showed the most prominent staining. CPC quantification in RGD-coated groups was also significantly higher than that in the control group. (**C**) Photographs of mineral nodules formed after 29 and 31 days of culture in the RAD-coated groups. (**D**) CPC quantification of mineralized product staining intensity in (**B**). The RAD-coated groups showed calcific deposition like the control group on both days 29 and 31. CPC data also depict the same results. *n* = 3, different lowercase characters denote significant differences compared to the control (*p* < 0.05 vs. control).

To investigate the effect of RGD and RAD peptides on the induction of mineralization, ARS staining was performed on days 29 and 31. All three RGD groups induced matrix mineralization of hDPSCs compared to the control (Figure 6A,B). However, the RAD peptide groups exhibited mineralization comparable to that in the control group (Figure 6C,D). CPC quantification of ARS staining further showed significant differences between the RGD groups and control group in experiment one. The enhanced staining intensity compared to the control group was as follows: 12 times by RGD-1 and RGD-2, and 22 times by RGD-3 on day 29; 15 times by RGD-1, 13 times by RGD-2 and 23 times by RGD-3 on day 31.

### 3.6. Effects of MAPK Inhibitors

**Figure 7:**
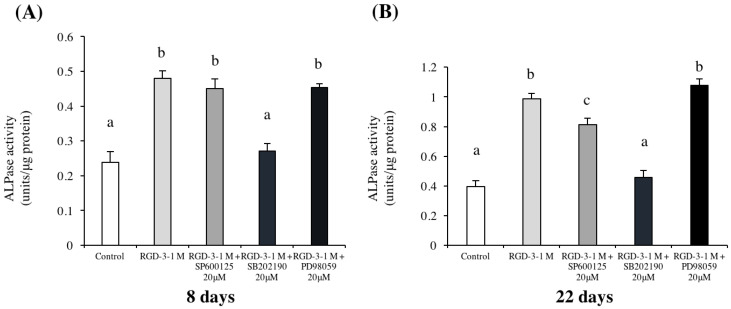
Relative ALP activity in response to MAPK pathway inhibitors. hDPSCs were spread onto non-TCPP plates coated with 1 M RGD-3 or dH_2_O (control) and were then exposed to 20 μM of one of three MAPK pathway inhibitors (SP600125 for JNK, SB202190 for p38, and PD98059 for ERK) on day 6 in serum-free DMEM for 2 h. Then, the medium was changed. ALP activity was assessed on days 8 (**A**) and 22 (**B**). p38 inhibitor neutralizes the effect of RGD-3 on hDPSC differentiation on both days. *n* = 3, different lowercase characters denote significant differences compared to the control (*p* < 0.05 vs. control).

In the ALP activity assay, the p38 inhibitor SB202190 neutralized the effect of RGD-3 to a level comparable to that in the control group on both day 8 and day 22 (Figure 7). The other inhibitors did not have any significant effect on day 8. On day 22, the JNK inhibitor SP600125 had a noticeable inhibitory effect, but much less prominent than the p38 inhibitor.

**Figure 8:**
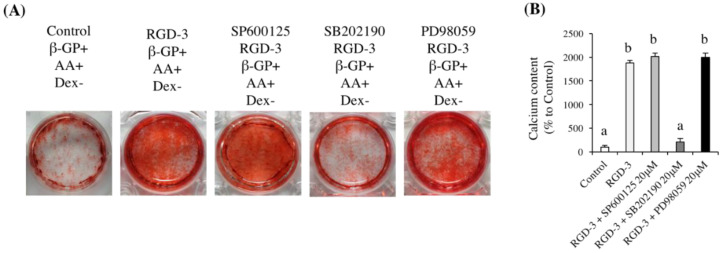
ARS staining in response to MAPK pathway inhibitors. hDPSCs were spread on non-TCPP plates coated with 1 M RGD-3 or dH_2_O (control) and were then exposed to 20 μM of one of three MAPK pathway inhibitors (SP600125 for JNK, SB202190 for p38 and PD98059 for ERK) on day 6 in serum-free DMEM for 2 h. Then, the medium was changed, and culture was continued. (**A**) Photographs of mineral nodules formed after 30 days of culture. (**B**) CPC quantification of mineralized product staining intensity in (**A**). p38 inhibitor neutralizes the effect of RGD-3 on hDPSC mineralization. *n* = 3, different lowercase characters denote significant differences compared to the control (*p* < 0.05 vs. control).

In ARS staining experiment, the p38 inhibitor reduced the matrix mineralization of hDPSCs and showed results comparable to the control, while the other two inhibitors induced the same mineralization as that observed in the RGD-3-coated group (Figure 8). CPC quantification data further supported this finding.

## 4. Discussion

In the present study, the significance of RGD peptides in DPP sequence were observed in terms of differentiation and mineralization and the mechanism behind their effects was explored as well. The effects of RGD peptides were investigated by dissolving the RGD peptide powders with dH_2_O and physically coating non-TCPS. The surfaces were left unwashed after the coating was dried, leaving all the peptides on the surfaces. Afterwards, hDPSCs were grown on the coated surfaces and their responses were observed. There was a noticeable difference in the number of cells attached on to the surfaces, starting from day one. This supports the previous statements from multiple studies that the RGD contains cell attachment properties, which may have played an active role in attracting more cells to the surface and eventually spreading out the cells faster. According to a study [19], initial cell attachment rate is an indication of matrix mineralization. Thus, if human DPP-derived RGD peptides were to be included in future pulp capping materials, it could act as an attractor of odontoblasts in the site of exposed pulp and aid in a quicker wound healing.

The RGD peptides promoted the differentiation of hDPSCs, observed by the promotion of ALP activity and enhanced mRNA expressions of odontogenic genes *DMP-1*, *DSPP*, *ALP* and *BSP*. Among the three RGD peptides, RGD-3 induced the strongest ALP activity on all days of the experiment, the highest value on day 22 compared to the control. The isolated RNA samples showed significantly upregulated mRNA expressions in all three RGD peptides, specifically RGD-3 compared to the control. Related results were observed in mineralization experiment where the RGD-3 had the most prominent calcific stain on both days 29 and 31. One probable reason for this result could be that RGD-3 contains a lysine residue at the C-terminus (SRGDASYNSDESKD) in its amino acid chain. A study compared different peptides including RGD, and observed their attachment to self-assembled monolayers. Among the different flanking sequences, lysine-containing RGDSPK performed better than the other three peptides [20]. In a further study, RGDSPK induced the highest level of ALP activity and mineralization of MC3T3-E1 osteoblastic cells [21]. Based on these studies, it was suggested that the extra amine in the side chain of lysine becomes positively charged and nonspecifically interacts with the negatively charged glycocalyx on the cell surface. Thus, the specific binding of RGD-3 to cell integrins is supplemented by the nonspecific binding of lysine residues to the cell membrane, which explains the importance of the specific conformation of RGD-3. As a result, cell adhesion and subsequent differentiation and mineralization were enhanced in the RGD-3 group.

The RAD groups were not significantly different than the dH_2_O-coated control in differentiation and mineralization, which suggests the importance of the RGD domain and the specific conformation of RGD-3 in the DPP sequence, which may play a predominant role in extracellular matrix-mediated signalling and the differentiation of dental pulp stem cells into odontoblasts. Moreover, all three RGD-peptides induced enhanced ALP activity and led to strong calcific staining compared to the control, which may be related to their individual conformation in the DPP sequence. To better understand their mechanism and effective clinical application, further study is required.

In continuation of the effects of human DPP-derived RGD peptides on hDPSCs, it was necessary to examine exactly how the signals transferred from extracellular space in to the cells, and if the RGD domain was involved in this process. That is where integrins and cell-signalling pathways come into discussion. Integrins, the cell adhesion receptors can transfer signals in and out of cells. Most integrins recognize multiple ECM proteins, and the specific configuration of their α and β structural subunits dictates the binding specificity and signalling properties of an individual integrin [22]. There are different cell signal transduction pathways for integrins. The MAPK pathways are established signal-transduction pathways [23,24]. There have been reports that these pathways become activated during tertiary dentinogenesis stage [25]. The three primary members of the protein kinase cascades in the MAPK pathways have vastly diverse regulatory domains, which enables them to respond to a wide range of stimulations [26]. However, the fact that a single specific MAPK protein is activated in response to a specific ECM protein is associated with scaffolding or anchoring proteins. The MAPK pathways have been linked to the SIBLING family of proteins as the pathway for elucidating specific cellular responses. In the present study, three specific inhibitors were chosen for the three main MAPK pathways: the highly selective SP600125 inhibitor of JNK1/2/3 (Jun amino-terminal kinase), the cell permeable SB202190 inhibitor of p38 α and β subunits which lack the ability to impact other protein kinases; and lastly PD98059 that blocks ERK (extracellular signal regulated kinase) activation [27]. It has been observed that Dex plays a role in promoting the differentiation and mineralization of rat odontoblast-like cells through MAPK pathway [28], so to observe the unbiased effect, Dex was not present in the mineralization-inducing medium. Based on the results of the present study experiments, RGD-3 was selected for the experiments regarding MAPK inhibitors, and an ALP activity assay and Alizarin red S staining were performed. The inhibition of p38 signalling had an evident effect in suppression of the differentiation and mineralization, which were accelerated multiple folds by RGD-3. The ALP activity was reduced to a level comparable to the control, starting as early as day 8. Interestingly, inhibition of JNK pathway had also reduced ALP activity on day 22 but unlike the complete inhibitory effect of p38 inhibitor, the JNK inhibitor had partial inhibitory effect.

The communication between cell-surface integrins and ECM proteins is crucial to mediating signals inside of the cells. In a study by Jadlowiec et al. [13], it was reported that phosphophoryn might function in osteoblastic gene expression by binding to certain αβ integrin receptors on the cell surface. Runx-2 mRNA expression was investigated and in response to an integrin-blocking α_v_β_3_ antibody, the mRNA expression of Runx-2 decreased by almost 60% in human mesenchymal stem cells. In the present study, RGD-3 triggered the upregulation of *ITGA1-5*, *ITGA7*, *ITGB1* and *ITGB3* compared to the control. Based on the remarkable upregulation in the present study, there is a possibility that integrins *ITGA5*, *ITGA7*, *ITGB1* and *ITGB3*, either alone or in combination, might be involved in intracellular signalling in hDPSCs.

## 5. Conclusions

The findings of the present study indicate that human DPP-derived RGD peptides, RGD-1, RGD-2 and RGD-3 promote the proliferation, differentiation and mineralization of hDPSCs in vitro. All three RGD peptides significantly induced ALP activity, ARS staining and promoted the mRNA expression of odontogenic genes. Among the RGD peptides, RGD-1 and RGD-2 had the same potential for differentiation. However, RGD-3 had the most prominent result. Investigation of the effects of certain cell signalling pathway inhibitors revealed that p38 inhibitor SB202190 was most effective among the MAPK inhibitors in differentiation and mineralization experiments. The mRNA expressions of integrins showed significant promotion of most of the alpha and beta subunit genes of integrin. It is possible that RGD-3 binds to the integrin receptors on the surface of hDPSCs and regulates differentiation gene expression via activation of p38 in the MAPK pathway. RGD-3 (SRGDASYNSDESKD) may be a promising material to be considered in future vital pulp therapy, and the inclusion of RGD-3 in the formulation of a novel pulp capping agent may induce undifferentiated pulp cells into odontoblasts and form reparative dentin in the exposed pulp.

## Figures and Tables

**Table 1:** Human primer information for real time RT-PCR.

Target Gene	Forward	Backward	Product Size (bp)	Annealing Temperature (°C)
*hGAPDH* (NM_001289746.2)	CACTAGGCGCTCACTGTTCTCT	CGTTCTCAGCCTTGACGGT	250	66
*hDMP-1* (NM_001079911.3)	ACAGCAGCTCAGCAGAGAGT	TAATAGCCGTCTTGGCAGTC	235	62.8
*hDSPP* (NM_014208.3)	TGCTGGCCTGGATAATTCCG	CTCCTGGCCCTTGCTGTTAT	136	66
*hALP* (NM_001127501.4)	ATGGGATGGGTGTCTCCACA	CCACGAAGGGGAACTTGTC	108	59.9
*hRunx-2* (NM_001015051.4)	TCGGAGAGGTACCAGATGGG	CTGTCTGTGCCTTCTGGGTT	263	59.9
*hBSP* (NM_004967.4)	AAGGGCACCTCGAAGACAAC	CCCTCGTATTCAACGGTGGT	119	62.8
*hOPN* (NM_001040058.2)	AGCAGCAGGAGGAGGCAGAGCA	TAACCTTTTTTACTGCCTA	314	59.9

**Table 2:** Human primer information for real time RT-PCR.

Target Gene	Forward	Backward	Product Size (bp)	Annealing Temperature (°C)
*hGAPDH* (NM_001289746.2)	CACTAGGCGCTCACTGTTCTCT	CGTTCTCAGCCTTGACGGT	250	66
*hITGA1 * (NM_181501.1)	CTCACTGTTGTTCTACGCTGC	ACGACTTGAAATGTGGGGCT	419	59.9
*hITGA2 * (NM_002203.3)	GTGGCTTTCCTGAGAACCGA	GAAGCTGGCTGAGAGCTGAA	278	62.8
*hITGA3* (NM_002204.3)	ATGGCAAGTGGCTGCTGTAT	GCACTCTAGCCACACACAGT	272	59.9
*hITGA4* (NM_000885.5)	AATCCCGGGGCGATTTACAG	TCCAGCTTGACATGATGCAAAA	354	59.9
*hITGA5* (NM_002205.4)	CCCTCATCTCCGGGACACTA	ATCCAACTCCAGGCCCTTTG	397	56.3
*hITGA6* (NM_000210.3)	CTCGCTGGGATCTTGATGCT	TCAGATGGCTGAGCATGGAT	128	59.9
*hITGA7* (NM_002206.2)	GGAAGACCGACAGCAGTTCA	ATCTTGATGCGACACCAGCA	264	59.9
*hITGA8* (NM_003638.2)	GCCTATGCCGAGTTCTCTCC	CCCAGTAAACTCCCCAGCAG	297	59.9
*hITGB1* (NM_002211.3)	GCCGCGCGGAAAAGATGAAT	TGCTGTTCCTTTGCTACGGT	323	59.9
*hITGB3* (NM_000212.2)	GAAGCAGAGTGTGTCACGGA	ACATGACACTGCCCGTCATT	201	59.9

## Data Availability

The data that support the findings of this study are available from the corresponding author upon reasonable request.
